# Dual-Drug Containing Core-Shell Nanoparticles for Lung Cancer Therapy

**DOI:** 10.1038/s41598-017-13320-4

**Published:** 2017-10-16

**Authors:** Jyothi U. Menon, Aneetta Kuriakose, Roshni Iyer, Elizabeth Hernandez, Leah Gandee, Shanrong Zhang, Masaya Takahashi, Zhang Zhang, Debabrata Saha, Kytai T. Nguyen

**Affiliations:** 1 Bioengineering Department, University of Texas at Arlington, Arlington, TX 76019 USA; 2 Graduate Biomedical Engineering Program at UT Southwestern Medical Center at Dallas, Dallas, TX 75390 USA; 3 Department of Urology at UT Southwestern Medical Center, Dallas, TX 75390 USA; 4 Advanced Imaging Research Center at UT Southwestern Medical Center, Dallas, TX 75390 USA; 5 Department of Radiation Oncology at UT Southwestern Medical Center, Dallas, TX 75390 USA; 6 Simmons Comprehensive Cancer Center at UT Southwestern Medical Center, Dallas, TX 75390 USA

## Abstract

Late-stage diagnosis of lung cancer occurs ~95% of the time due to late manifestation of its symptoms, necessitating rigorous treatment following diagnosis. Existing treatment methods are limited by lack of specificity, systemic toxicity, temporary remission, and radio-resistance in lung cancer cells. In this research, we have developed a folate receptor-targeting multifunctional dual drug-loaded nanoparticle (MDNP) containing a poly(N-isopropylacrylamide)-carboxymethyl chitosan shell and poly lactic-co-glycolic acid (PLGA) core for enhancing localized chemo-radiotherapy to effectively treat lung cancers. The formulation provided controlled releases of the encapsulated therapeutic compounds, NU7441 - a potent radiosensitizer, and gemcitabine - an FDA approved chemotherapeutic drug for lung cancer chemo-radiotherapy. The MDNPs showed biphasic NU7441 release and pH-dependent release of gemcitabine. These nanoparticles also demonstrated good stability, excellent hemocompatibility, outstanding *in vitro* cytocompatibility with alveolar Type I cells, and dose-dependent caveolae-mediated *in vitro* uptake by lung cancer cells. In addition, they could be encapsulated with superparamagnetic iron oxide (SPIO) nanoparticles and visualized by MRI *in vivo*. Preliminary *in vivo* results demonstrated the low toxicity of these particles and their use in chemo-radiotherapy to effectively reduce lung tumors. These results indicate that MDNPs can potentially be used as nano-vehicles to provide simultaneous chemotherapy and radiation sensitization for lung cancer treatment.

## Introduction

Lung cancer is a leading cause of cancer-related mortality in the United States with a quarter of all cancer-related deaths in 2015 attributed to this disease^1^. A significant number of diagnosed patients usually present advanced non-small cell lung cancer (NSCLC) due to its asymptomatic nature, thereby necessitating rigorous and specific treatment upon diagnosis^2,3^. Combination drug therapies are being explored, as the synergistic action of multiple drugs could potentially lead to better therapeutic efficacy and reduced possibility of drug resistance development by the cancer cells^4^. Gemcitabine-platinum and cisplatin-gemcitabine-bevacizumab are some of the drug combinations currently in clinical trials for the treatment of advanced NSCLC^5,6^. Gemcitabine, an FDA-approved chemotherapeutic drug, has already been established for first-line chemotherapy treatment of NSCLC and used in clinical trials in combination with radiotherapy^7^. However, these conventional treatments are limited by rapid relapse^8^, systemic toxicity, and non-specific delivery of the drug combinations used, leading to low therapeutic efficacy^9,10^. Poor overall survival rates in NSCLC patients may also be attributed to intrinsic radiation resistance due to the increased ability of cancer cells to repair DNA damage after radiation therapy (RT)^11,12^. Therefore, it is crucial to develop a system that can overcome the above limitations and provide targeted and controlled releases of these therapeutic reagents for effective chemo-radiation therapy to treat these lung cancer patients.

Nanomedicine has recently gained widespread attention for cancer treatment due to improved drug solubility and bioavailability, increased site-specific targeting, reduced systemic toxicity, and incorporation of multiple components that impart multi-functionality for diagnosis and therapy^11,13^. Stimuli-responsive or ‘smart’ polymer-based nanoparticle systems can undergo rapid, reversible phase transitions in response to external stimuli, thereby releasing their contents at the site of interest “on demand.”^14^ Due to these advantages, we proposed to develop a stimuli-responsive core-shell nanoparticle system conjugated with folic acid and containing two different drugs, for targeted combination treatment of lung cancer. In this research, poly(N-isopropylacrylamide) (PNIPAAm) was copolymerized with biodegradable carboxymethyl chitosan (CMC) to form a semi-interpenetrating network (IPN) to impart both pH- and temperature-sensitivity and degradability to the final product by cleavage of CMC-PNIPAAm bond. PNIPAAm and chitosan are two commonly used thermo-responsive and pH-responsive polymers, respectively^15^. The method of polymerization of PNIPAAm and CMC has been done previously to form IPN hydrogels^16,17^. The PNIPAAm-CMC combination formed the shell of the nanoparticle (NP) system while the core consisted of poly lactic-co-glycolic acid (PLGA) and imaging contrast agents such as iron oxide. The PLGA allows controlled release of the encapsulated compounds while superparamagnetic iron oxide (SPIO) can be used for Magnetic Resonance Imaging (MRI) and to induce temperature changes using an external alternating magnetic field for hyperthermia therapy or radiofrequency ablation, if needed^18–20^. These temperature changes can facilitate temperature-responsive burst release of gemcitabine from the PNIPAAm-CMC shell for more accurate therapy.

In order to radiosensitize the cancer cells, our PLGA core was encapsulated with 8-dibenzothiophen-4-yl-2-morpholin-4-yl-chromen-4-one (NU7441), a highly potent radiosensitizer known to selectively inhibit DNA-dependent protein kinase (DNA-PK). Following DNA-PK inhibition, cells are unable to repair DNA damage following radiotherapy and eventually die through various mechanisms^21^. The PNIPAAm-CMC shell contained gemcitabine, which would release rapidly in response to the acidic tumor environment (low pH) to effectively kill cancer cells. Furthermore, the MDNPs were surface conjugated with folic acid to actively target the folate receptor-α known to be overexpressed in a number of human cancer cells including lung cancer cells^22^. The glycosylphosphatidylinositol-anchored folate receptor is known to show high affinity for folic acid, which it usually captures to feed the fast-dividing cancer cells^23–25^. Therefore, the innovation of this project lies in the development of a novel biodegradable multi-functional nanocarrier that can actively target folate receptor-overexpressing lung cancer cells followed by the release of two different therapeutic agents for non-invasive and effective lung cancer chemo-radiotherapy.

## Results

### Characterization of MDNPs

**Figure 1:**
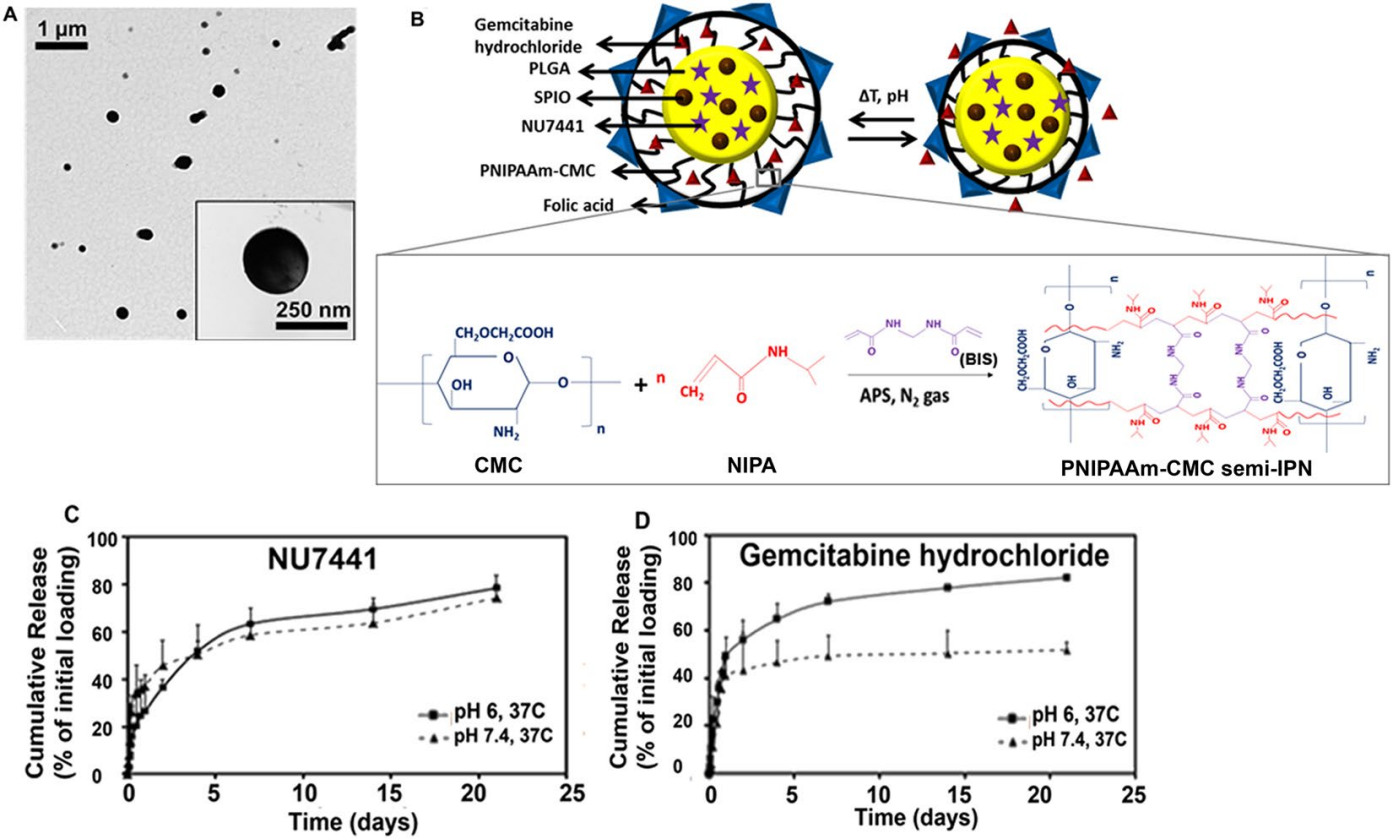
Characterization of MDNPs. (**A**) TEM image of MDNPs showing smooth spherical morphology (250–280 nm). (**B**) Schematic representation of the stimuli-sensitive behavior of MDNPs and the structure of the PNIPAAm-CMC semi-IPN shell. (**C**) Controlled bi-phasic release of NU7441 was observed from the PLGA core at 37 °C. (**D**) Gemcitabine hydrochloride in the PNIPAAm-CMC shell showed pH-dependent release with the highest release at pH 6 and 37 °C (n = 4).

Incorporation of all components in the final nanoparticle (NP) system was confirmed by FTIR (Supplementary Section, Fig. S1). The optimal ratio and concentration of polymers, drugs and targeting ligand (folic acid) used was determined using Stat-Ease for Design of Experiments (DoE). The morphology and schematic of the final MDNP system has been shown in Fig. 1A,B. Dynamic light scattering readings indicate that MDNPs had a hydrodynamic diameter of 289 ± 49 nm. The MDNPs were relatively well-dispersed and stable (polydispersity: 0.32, zeta potential: −36 mV) (Supplementary Section, Table 1). TEM images established that MDNPs were in the 250–280 nm size range and had smooth, spherical morphology (Fig. 1A).

Drug release kinetics of the MDNPs was studied at 37 °C and different pH conditions - pH: 7.4 (physiological pH) and 6.0 (approx. pH prevalent in an acidic tumor microenvironment). The loading efficiencies for NU7441 and gemcitabine hydrochloride were 52% and 88%, respectively. The release of NU7441 from the PLGA core was bi-phasic with a burst release of 30% of encapsulated NU7441 within a day and a sustained release of about 70% after 21 days of incubation (Fig. 1C). The gemcitabine hydrochloride loaded into the PNIPAAm-CMC shell showed pH-dependent release of 82% of incorporated gemcitabine at pH 6 and 52% at pH 7.4 at 37 °C, at a period of 21 days after incubation (Fig. 1D).

The MDNPs were stable and showed a 63% decrease in weight by day 27 of the degradation study at 37 °C (Supplementary Section, Fig. S1B,C). The MDNPs were also temperature and pH-responsive, as shown in the Supplementary Section, Fig. S1D,E. The incorporation of SPIO within the particles and the magnetic and imaging properties of MDNPs were confirmed using SQUID magnetometry and MRI (Supplementary Section, Fig. S1F,E).

### *In vitro* Cell Studies

**Figure 2:**
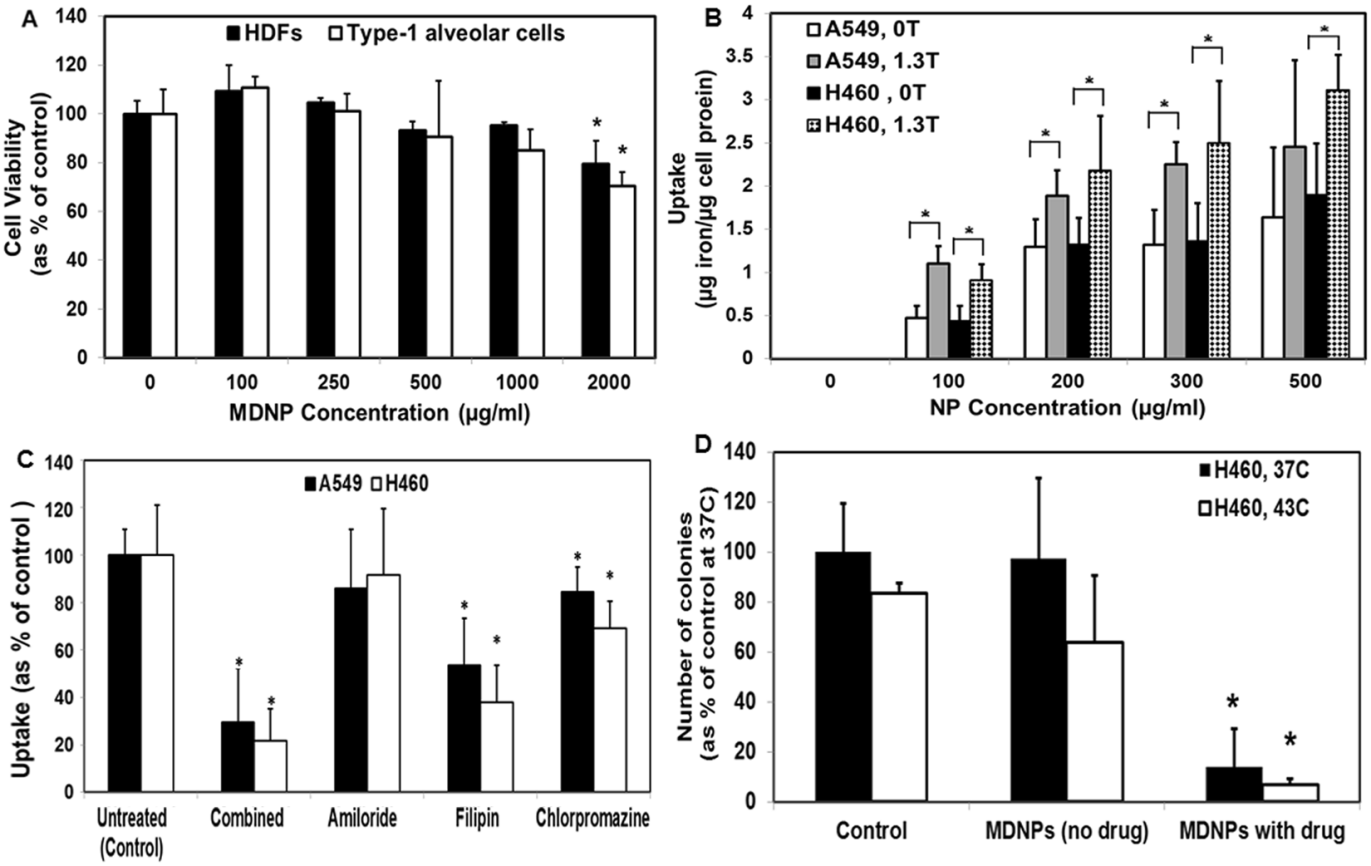
*In vitro* testing studies of MDNPs. (**A**) Good MDNP cytocompatibility with HDFs and AT-1 cells up to 1 mg/ml concentration, demonstrated using MTS assays (n = 4, *p < 0.05 w.r.t cell viability at 0 µg/ml MDNP concentration). (**B**) *In vitro* MDNP uptake by A549 and H460 lung cancer cells was dependent on MDNP dose (n = 4, *p < 0.05). T = Tesla (**C**) Mechanism of uptake studies show a significant reduction in MDNP uptake in cells treated with filipin, an inhibitor of caveolae-mediated endocytosis (n = 4, *p < 0.05 w.r.t untreated control group). (**D**) Colony forming study indicates that empty MDNPs did not have significant effects on H460 and A549 cells. Drug-loaded MDNPs significantly reduced colony formation (n = 4, *p < 0.05 w.r.t controls at respective temperatures).

The presence of folate receptors on A549 and H460 lung cancer cells was confirmed by Western blot and Resonant Sensors Inc. (RSI) Bioassay system (Supplementary Section, Fig. S2). MTS assay results indicate that the non-drug-loaded MDNPs showed cytocompatibility with human dermal fibroblasts (HDFs) and Type-1 alveollar (AT1) cells up to 1 mg/ml concentration following 24 hours’ incubation. At all concentrations, more than 80% of the cells were viable, indicating that the particles are relatively non-toxic (Fig. 2A). These results were confirmed using DNA assays (Supplementary Section, Fig. S3A). Dose-dependent uptake of MDNPs by A549 and H460 lung cancer cells was observed *in vitro* (Fig. 2B). Uptake was observed to be greater in the presence of an external magnet (1.3 Tesla) than in the absence of the magnet (0 T) indicating that application of an external magnetic field may be useful for greater MDNP uptake by cancer cells, which would in turn result in better treatment due to the accumulation and subsequent release of greater amount of the therapeutic agents within the cells. There was, however, a comparatively lower dose-dependent uptake of MDNPs by AT1 and human bronchial epithelial cells (HBECs) than by lung cancer cells (Supplementary Section, Fig. S3B). In addition, cell activation studies indicated that the particles do not promote significant production of inflammatory cytokines by the AT1 cells *in vitro* following internalization (Supplementary Section, Fig. S3C,D). A mechanism of uptake study was also conducted to determine the route of nanoparticle uptake in A549 and H460 lung cancer cells. As shown in Fig. 2C, MDNP uptake was significantly reduced when A549 and H460 cells were treated with filipin – an inhibitor of caveolae-mediated endocytosis(40% and 55% reduction, respectively, compared to untreated cells). A significant decrease in uptake was also observed in cells treated with chloropromazine, known to inhibit clathrin-mediated endocytosis. However, greater inhibition of MDNP uptake occurred on cells treated with filipin than that with chlorpromazine. Based on the Western blot, RSI system, and uptake results, H460 cells showed greater uptake of MDNPs and hence, were chosen for further *in vitro* and *in vivo* studies.

### *In vitro* Clonogenic Studies

To assess the therapeutic efficacy of MDNPs, a colony forming study was conducted as previously described^26^. No significant decrease in H460 and A549 colony numbers was observed on incubation with empty MDNPs when compared to the controls (untreated cells). MDNPs loaded with gemcitabine and NU7441 showed significant reduction in colony formation to 14% compared to that of the controls. The empty MDNPs did not impact cell viability or colony formation (Fig. 2D).

### Hemocompatibility Studies

**Figure 3:**
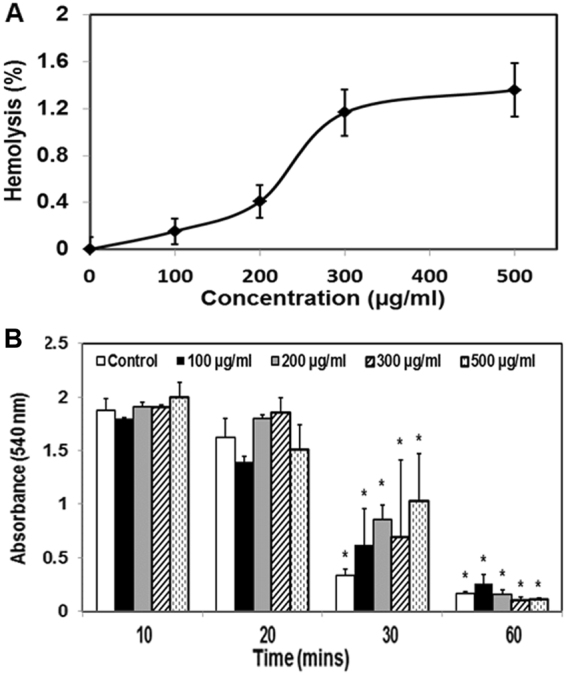
Hemocompatibility of MDNPs. (**A**) Less than 2% hemolysis occurred at MDNP concentration of 500 µg/ml indicating that the particles are non-hemolytic. (**B**) Blood clotting on exposure to varying MDNP concentrations occurred at the same rate as the control (blood not exposed to MDNPs) (n = 9, *p < 0.05 w.r.t absorbance readings at 10 minutes).

Hemolysis study results showed that less than 2% hemolysis occurred even at 500 µg/ml MDNP concentration, indicating that these particles are non-hemolytic (Fig. 3A). Visual observation also showed reddening of the solution for the positive control, indicating hemolysis, while no visual indication of hemolysis could be seen for the negative control and nanoparticle groups (blood exposed to 100, 200, 300, and 500 μg/ml MDNP concentration) (Supplementary section, Fig. S4A). Furthermore, studies were conducted to determine if the MDNPs would alter the clot formation upon entering the blood stream after administration. As seen in Fig. 3B, the absorbance values for all experimental samples decreased gradually, indicating clot formation in the samples aligning with time. The MDNPs at varying concentrations ranging from 100 to 500 µg/ml showed the same blood clotting rate as the control group (whole blood not exposed to NPs). This was visually confirmed as the solution for the control group and 500 µg/ml group had comparatively clearer supernatants at 60 minutes than at other time points. The clot formation is clearly visible in both experimental groups during the 30 and 60 minute time points (Supplementary section, Fig. S4B).

### *In vivo* Investigation of MDNPs

#### *In vivo* Imaging

**Figure 4:**
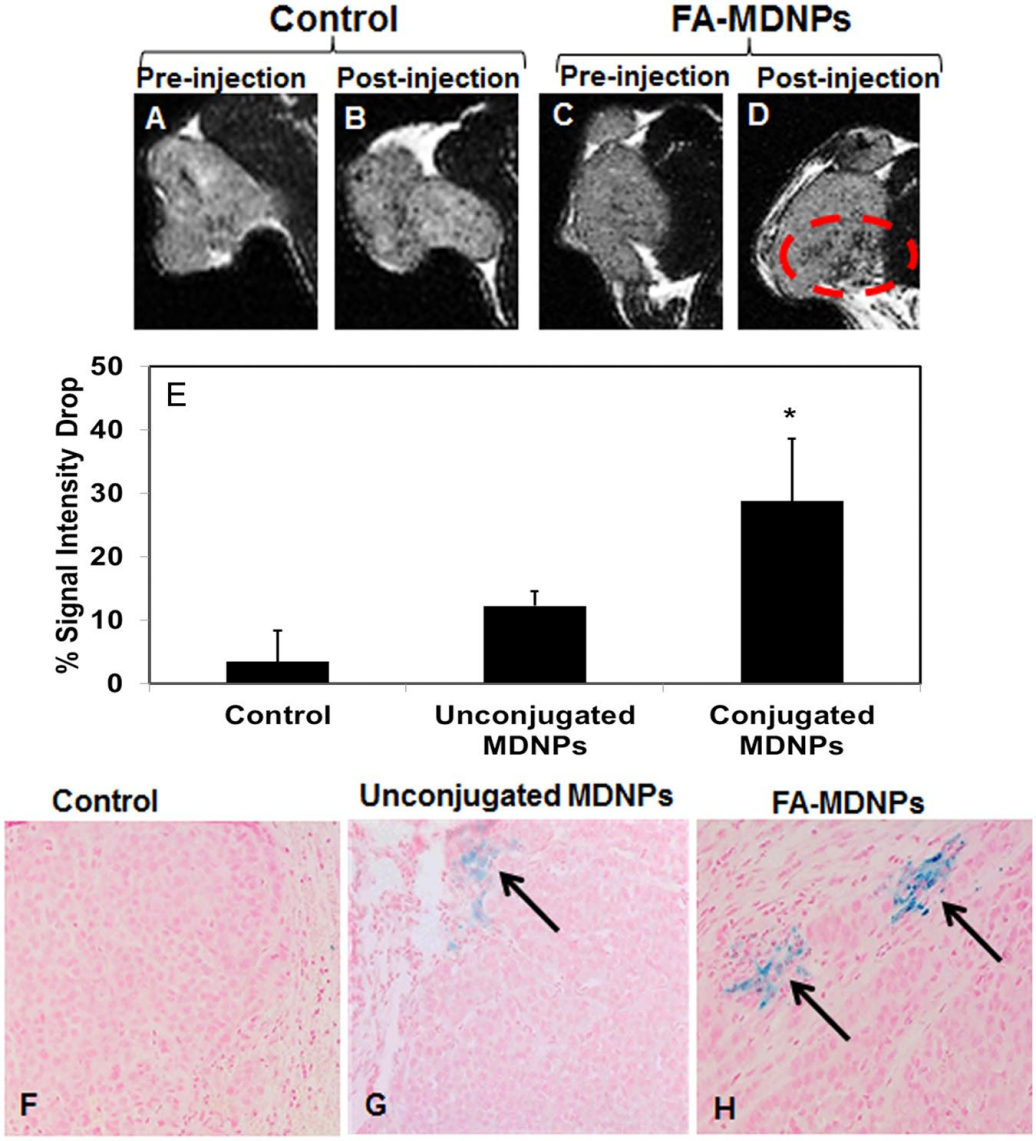
*In vivo* MRI potential of MDNPs. (**A**,**B**,**C**,**D**) MRI images of the tumors in the control group and the group treated with folic acid-conjugated MDNPs (FA-MDNPs) before and after injection of the respective solutions. A distinct darkening of the tumor was observed in the group treated with MDNPs post injection. (**E**) Significant T2 signal intensity drop was observed in the case of FA-MDNPs indicating greater negative contrast due to the presence of iron oxide in the tumor (n = 4). (**F**,**G**,**H**) Prussian blue staining on the tumors (10x magnification). More blue regions (arrows) seen in the FA-MDNPs group indicating presence of greater amount of iron oxide in the tumor.

Tumors in H460 tumor bearing mice were visualized by MRI before and 24 hours after injection of the MDNP suspension. The darkening of the tumor region was seen 24 hours after the injection of the folic acid-conjugated MDNPs (Fig. 4A,B,C,D). T2 signal intensity in the tumors was found to drop significantly by 30% in animals treated with folic acid-conjugated MDNPs compared to the tumor signal intensity before treatment. On the other hand, control (untreated) animals and the animals treated with unconjugated MDNPs showed signal intensity drops of about 3.5% and 12%, respectively (Fig. 4E). In addition, more iron could be seen in tumor sections treated with folic acid-conjugated MDNPs compared to sections treated with unconjugated MDNPs using Prussian blue staining (Fig. 4F,G,H) 24 hours post-treatment. This indicates that the folic acid-conjugated MDNPs were retained longer in the tumor following their administration.

#### *In vivo* Therapeutic Efficacy

**Figure 5:**
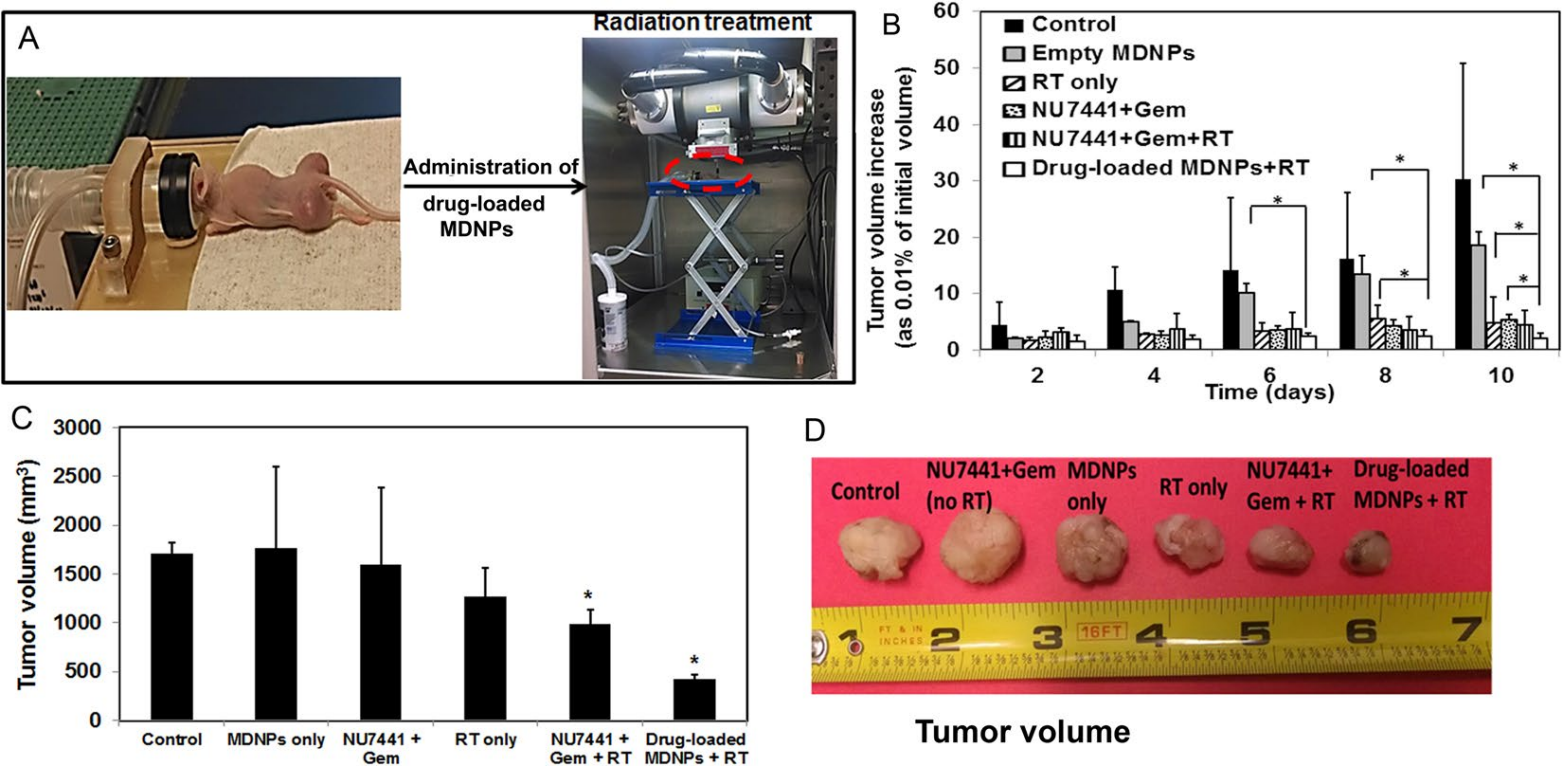
*In vivo* therapeutic efficacy of MDNPs. (**A**) Representative images showing the anesthesia set up for the *in vivo* studies and the set up for radiation treatment. The circled area shows the mouse under anesthesia inside the X-rad320 small animal irradiator. (**B**) Changes in tumor volume for each group as the percentage of the initial tumor volume mesured in the beginning of the treatment study. Tumor volumes on day 0 were assumed to be 100%. Significantly slower tumor growth rate was observed in the case of the ‘NU7441 + Gem + RT’ group and the ‘drug-loaded MDNPs + RT’ group compared to other treatment groups at days 6, 8, and 10 (n = 4, *p < 0.05 for ‘drug-loaded MDNPs + RT’ group compared to other treatment groups). (**C**) *Ex vivo* tumor volumes of the different treatment groups at day 12 showing much smaller tumor size of the ‘drug-loaded MDNPs + RT’ group compared to the other groups. (**D**) Representative images of tumors from the different treatment groups.

Furthermore, the efficacy of drug-loaded MDNPs was studied in H460-tumor bearing athymic nude mice, which are commonly used in preliminary *in vivo* cancer research to study the therapeutic efficacy of the investigational compounds/ drug carriers. For the groups requiring irradiation of the tumors, radiation therapy (RT) was provided 24 hours after treatment, and the setup for RT is shown in Fig. 5A. In this preliminary proof-of-principle study, tumors in the control (sham) group grew exponentially compared to their initial volume within 12 days. Tumors in the ‘drug cocktail’ (NU7441 + Gemcitabine) were found to be significantly smaller than the control group. The tumor inhibitory effect of the drug cocktail can be attributed to the therapeutic effect of gemcitabine hydrochloride. A significant inhibition of tumor growth was observed in the ‘drug cocktail + RT’ group and ‘drug-loaded MDNPs + RT’ groups (Fig. 5B). However, the tumor volume of groups treated with drug-loaded MDNPs + RT were smaller than the tumor volumes of groups given the drug cocktail (gemcitabine + NU7441) and RT alone. Following treatment, the animals were sacrificed and the tumors excised for *ex vivo* volume measurements (Fig. 5C) and visual observation (Fig. 5D). It was clearly shown that tumors of animals treated with drug loaded particles + RT were the smallest compared to those of other studied groups at the end of the study. Our preliminary results thus indicate the potential of our MDNPs as carriers of therapeutic agents for combined therapies such as chemo-radiotherapy to effectively treat lung cancers.

#### Histopathological Assessment of MDNP Toxicity *In vivo*

**Figure 6:**
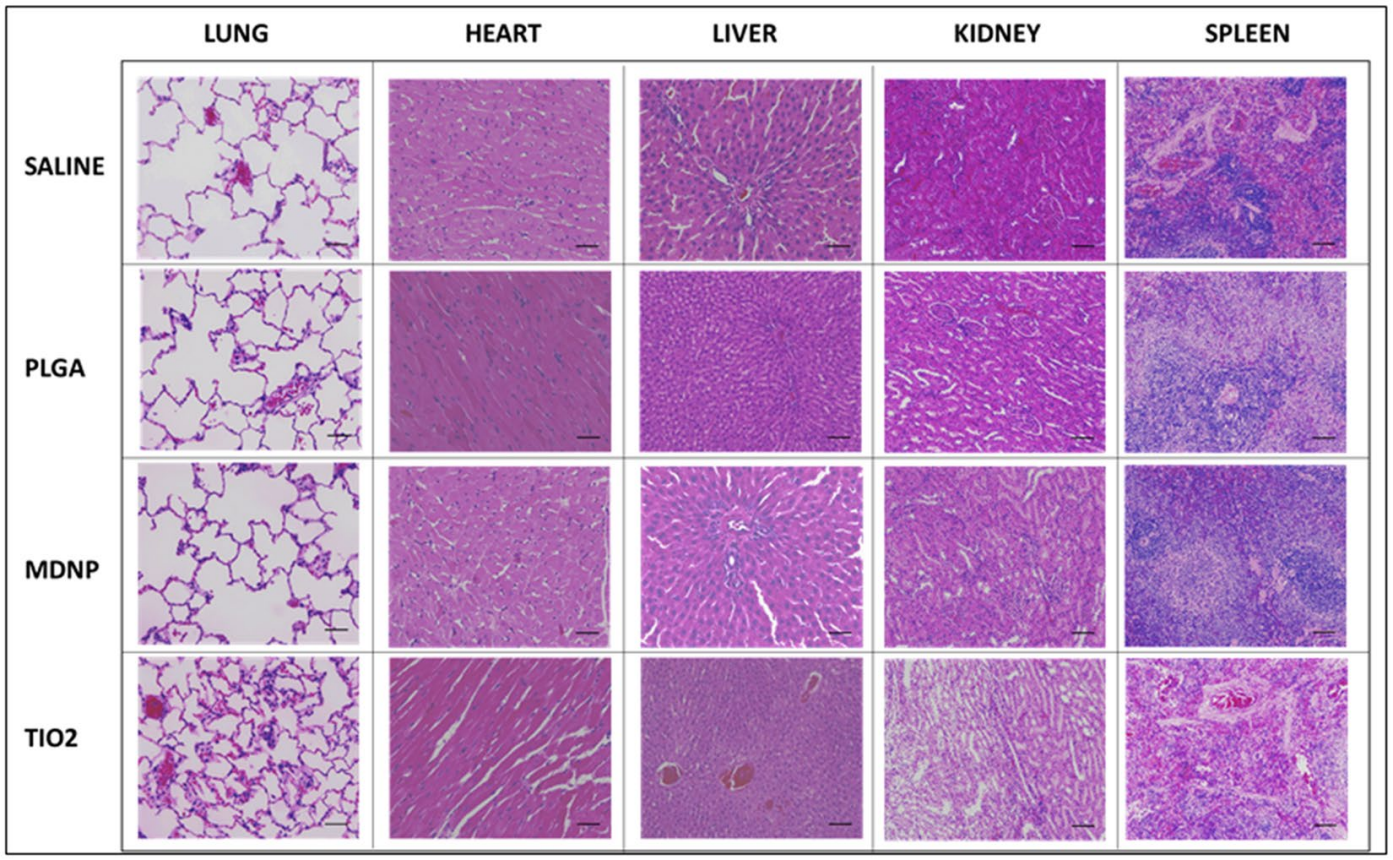
Safety of MDNPs *in vivo*. Histopathological examination of rat tissues treated with non-drug loaded MDNPs 7 days after exposure. The photomicrographs do not reveal any evidence of toxicity from the nanoparticles and were observed to be similar to that of the saline group (n = 3, Scale = 100 µm).

The histological micrographs of lung, heart, liver, kidney and spleen specimens collected from animals post 7 days of exposure to 0.9% saline, bare PLGA, bare MDNPs or TiO_2_ by nebulization are depicted in Fig. 6. We observed several evidences of toxicity in the group exposed to TiO_2_ nanoparticles, which are well-known as toxic nanomaterials, such as cell infiltration, septal thickening and collapsed airways. The liver and spleen samples from the TiO_2_ group also revealed the presence of small lesions. On the other hand, there was no evidence of toxicity found in the specimens exposed to the MDNPs, and they were found to be similar to that of the negative control group and bare PLGA NPs.

## Discussion

We have developed an innovative core-shell nanoparticle system for the treatment of lung cancers by effectively combining chemo- and radio-therapy. This nanoparticle system has several attractive features. First, it consists of a PLGA core for controlled release of a potent radiosensitizer – NU7441 over 3 weeks and a pH-sensitive degradable polymer shell for controlled release of a chemo-drug – gemcitabine hydrochloride in response to the acidic tumor environment. Secondly, the potential incorporation of contrast agents, such as SPIO, in our nanoparticle system aids in *i* *n vivo* tracking of the particles by MRI. Thirdly, it allows simultaneous delivery of a chemotherapeutic agent (gemcitabine) and a radio-sensitizer (NU7441). Since gemcitabine is already FDA-approved for lung cancer treatment, we hypothesize that the combination of this drug with a potent DNA DSB repair inhibitor NU7441 would enhance its therapeutic efficacy to treat advanced-stage lung cancer through chemotherapy and radiotherapy. Finally, the presence of folic acid on the surface of the MDNPs will aid in targeting folate receptor-overexpressing lung cancer cells, thereby facilitating site-specific therapy.

The formed MDNPs (average diameter of 289 ± 49 nm) could be used as drug delivery systems for pulmonary delivery. Previous studies indicate that NPs in the 0.5–3 µm size range tend to be taken up by alveolar macrophages and cleared rapidly from the body, thereby reducing their effectiveness as pulmonary drug delivery systems^27^. Hence, particles with sizes less than 0.5 µm would be ideal for pulmonary drug delivery. PLGA-magnetic nanoparticles (293 nm size) developed by Verma *et al*.^28^. reached the lung tissue of female BALB/c mice following nebulization. Porous and non-porous voriconazole-containing PLGA NPs (~207–291 nm) developed by Sinha *et al*.^29^ were also observed in murine lung tissue up to 7 days, respectively following dry-powder inhalation. Based on results from previous studies, the MDNP size is within the range used for pulmonary drug delivery and will potentially reach the deep lung tissue following inhalation. FTIR results confirm that all the components used during synthesis, i.e., SPIO, PLGA, PNIPA, CMC and folic acid have been incorporated in the final system. The high zeta potential value of MDNPs demonstrates good colloidal stability because the electrostatic repulsion between the particles dominates van der Waals forces of attraction between them, thus ensuring they remain stable in solution^30^. The MDNPs also maintained their magnetic properties, and previous studies from others have demonstrated that particles with similar or lower magnetization have been successfully used for MRI and drug delivery^31^.

Following physical characterization, the pH-responsiveness of MDNPs was studied. A decrease in MDNP size from high to low pH is in keeping with findings by Li *et al*.^32^., whose chitosan-graft-PNIPAAm hollow spheres demonstrated significantly smaller sizes between pH 4.5–6.5 than at other pH conditions. This behavior was also observed with CS-PNIPAAm nanoparticles, which had smaller sizes from pH 2 to 5 and larger sizes at pH > 6^33^. A similar trend of shrinking at acidic pH (pH ~2) and swelling at basic pH (~7.4) has been observed in PNIPAAm-CMC hydrogels^16,17^. Nanoparticles responsive to pH changes have earlier been exploited to deliver drugs within the tumor microenvironment and/or intracellularly within the endosome or lysosome due to polymer protonation at a pH lower than the physiological pH (7.4)^34,35^. Due to the amphoteric nature of CMC (presence of both carboxyl and amine groups), the particles are expected to swell at acidic pH (pH < 2) due to protonation of the amino groups. However when the pH is higher than 2, the interaction between partially deionized COOH groups and partially deprotonated amino groups would lead to inter- and intramolecular hydrogen bond formation within the shell and associated hydrophobic interactions, which would cause shrinking of the particles^36,37^. At pH 7 and above, the network becomes hydrophilic because of ionization of –COOH groups, leading to swelling of the system once again^16,36,37^.

The MDNPs’ LCST of 43 °C differs from results by Kim *et al*. who observed an LCST of 35 °C for their PNIPAAm-chitosan graft copolymer and blend^38^. The use of CMC by us instead of chitosan, and the variation in monomer amounts used could cause the difference in LCST values between our nanoparticles versus their materials. The bi-phasic NU77441 release pattern from MDNPs is characteristic of PLGA NPs^39,40^. The release behavior was dependent on PLGA degradation and drug diffusion^41^. The pH-responsive drug release from the MDNPs shell concurs with our previous results using PNIPAAm-chitosan nanoparticles, which showed thermo- and pH-responsive drug release at 40 °C and pH 6 respectively^42^. Similar pH-sensitive behavior was also observed in chitosan-g-PNIPAAm nanoparticles prepared by Duan *et al*.^43^., which showed an increase in drug release from a 50% release at pH 7.4 to more than 80% release at pH 5.0–6.5 at 37 °C. This gradual release of both encapsulated agents will be useful for providing targeted, controlled drug delivery while overcoming systemic toxicity and the need for multiple dosages, which are the current limitations of conventional drug administration methods.

The MDNPs were found to be cytocompatible with low toxicity *in vitro* using cell cultures. Good MDNP cytocompatibility with HDFs and AT1 cells observed in our studies concurs with results from previous studies with the copolymer^44,45^ and nanoparticles prepared using the monomers (either PNIPAAm or carboxymethyl chitosan)^46,47^. The dose-dependent uptake seen by us is also in line with the findings of Sanoj Rejinold *et al*.^48^; they observed a concentration-dependent uptake of PNIPAAm-chitosan nanoparticles by PC3 prostate cancer cells. Our mechanism of uptake results are in accordance with previous findings which show that folic acid binds to folate receptors clustered around invaginated caveolae on the cell surface^49^. Our results (greater inhibition of uptake by filipin than chlorpromazine) also agree with findings by Li *et al*.^50^. where folate-decorated N-2-hydroxypropyl)methacrylamide copolymer – fluorescein isothiocyanate conjugates (FA-HPMA-FITC) were chiefly taken up by caveolae-mediated endocytosis in HeLa cervical cancer cells. We have previously demonstrated the *in vitro* potency of NU7441 encapsulated within prostate cancer-targeting PLGA NPs, where these particles showed a significant radio-sensitizing effect of PC3 prostate cancer cells compared to empty PLGA NPs^51^. Gemcitabine hydrochloride is a commonly used chemotherapeutic drug often prescribed as first line of therapy for lung cancer patients^7^ and is now being studied in combination with radiation therapy to provide better treatment via combination lung cancer therapy. Our clonogenic studies indicate that while the empty particles not containing drugs had no effect on colony survival, the MDNPs loaded with NU7441 and gemcitabine hydrochloride significantly reduced H460 and A549 colony survival to about 14% and 19% respectively compared to the untreated controls.

As part of our *in vitro* investigation, the inflammatory cytokine production by AT1 cells after MDNP exposure was studied. IL-6 is produced on exposure to acute air pollutants while IL-10 is a regulatory cytokine that decreases inflammatory responses by inhibiting production of other inflammatory cytokines^52^. IL-8 is released in response to airway inflammation although the mechanism is unknown^53^, while TNF-α is commonly produced by alveolar epithelial cells in the case of acute inflammation and injury^54^. Our results indicate negligible production of these cytokines by AT1 cells exposed to MDNPs, which agrees with previous works on PLGA based NPs that did not stimulate TNF-α production in the THP1 monocytic cell line even at a high concentration of 5 mg/ml^55^. In contrast, titanium nanoparticles have previously elicited large production of IL-1, IL-6, IL-8 and TNF- α by human bronchial epithelial cell lines^56^.

Due to the alveolar-capillary interface in the lungs, the hemolytic property of NPs should be studied extensively to ensure they do not lyse RBCs on entering the blood stream, which could result in critical consequences including anemia, jaundice, acute renal failure, and eventually death^57,58^. Studies have shown that formulations causing < 5% hemolysis are considered to be hemocompatible^48,57^. For example, suberoylanilide hydroxamic acid-loaded PLGA NPs showed only about 2% hemolysis of rat RBCs, indicating that they are hemocompatible^59^. Similarly, chitosan-g-PNIPAAm nanoparticles demonstrated 2% hemolysis and hence, were considered compatible with human blood^48^. Thus, the MDNPs can be considered to be hemocompatible as they only caused 2% hemolysis at a high concentration of 1 mg/ml.

Following *in vitro* characterization, preliminary *in vivo* investigation of MDNPs was carried out as a proof-of-concept to determine whether the MDNPs can be visualized by MRI *in vivo* and to test for toxicity following inhalation. Intratumoral injection was done in animal models of lung cancers by other research groups to mimic the localized therapeutic effect of NPs following inhalational drug delivery^10,60,61^. Our MRI results showing MDNP retention possibly via specific ligands such as folic acid in the tumor 24 hours post injection are in agreement with previous studies done by Wang *et al*.^62^., whose folic acid conjugated dendrimer-entrapped gold nanoparticles could be visualized in the lung tumor implanted in BALB/C nude mice 6 hours following administration. The accumulation in the lung tumor was observed for different routes of administration (intratumoral, intraperitoneal, and intravenous) in that study. Folate receptor-targeting nanomicelles for lung cancer therapy by dry powder inhalation have also been developed by Rosière *et al*.^63^. Similarly, a significantly higher accumulation of folate-conjugated Gadolinium (Gd)-based nanoparticles than PEG-conjugated Gd nanoparticles was also found in the folate receptor-expressing KB (human nasopharyngeal carcinoma) tumors of athymic mice 24 hours following tail vein injection^64^. These results indicate that conjugation of specific molecules such as folic acid could aid in the accumulation and retention of the nanoparticle system at the tumor site for a longer time than unconjugated nanoparticles.

Combined drugs and/or therapies using NPs have demonstrated better therapeutic effects compared to either systemic delivery of combined drugs or that of a single drug and/or therapy to treat cancers. For instance, the liposomal cisplatin-gemcitabine combination has already shown greater therapeutic efficacy and significantly lower nephrotoxicity during Phase II clinical studies in advanced NSCLC patients than a free cisplatin-gemcitabine combination^6^. NU7441 – the radiosensitizer included in our MDNP preparation, has demonstrated excellent *in vitro* and *in vivo* potency and DNA-PK inhabitation capabilities^21,65^; however, its poor water solubility, systemic toxicity, and reduced oral bioavailability affects its applicability in a clinical setting^65^. Therefore, incorporation of NU7441 in a nanocarrier can potentially overcome the above limitations, while providing controlled release of NU7441 at the targeted site for prolonged and effective radiosensitization. The significant slowing down of the lung tumor growth observed in animals treated with chemo-radiation therapy via MDNPs using the radiation-chemotherapy combination, NU7441 and gemcitabine, by us agrees with results of Rocca *et al*.^66^. In their study, a combination of radiation and cisplatin-containing polysilsesquioxane nanoparticles for chemo radiotherapy significantly inhibited A549 and H460 tumor growth in Nu/Nu lung cancer xenograft models, compared to other groups such as saline control, RT only, or cisplatin + RT. Similarly, preclinical evaluation of Genexol-PM, a nanoparticle formulation containing paclitaxel clinically approved in South Korea for radiosensitization of NSCLC, demonstrated good chemo-radio-therapeutic results *in vivo* ^67^. Nu/Nu lung cancer xenograft models showed a significant delay in tumor growth when treated with Genexol-PM and radiation therapy compared to groups given saline, RT only, or paclitaxel + RT. These results, in addition to our results, indicate the potential of combined chemo- and radiation-therapy and the use of nanoparticles as a drug carrier in lung cancer treatment.

To study the effect of MDNPs on the healthy lung, the particles were also delivered by nebulization to rats. Future studies with MDNPs will focus on the delivery of this nanoparticle system by inhalation to orthotopic lung tumors for maximum therapeutic efficacy, as the particles will have direct access to the diseased cells in the lungs while folate targeting will help in recognition of folate receptors overexpressed by lung cancer cells to potentially provide lung cancer-specific treatment. This is why nebulization was chosen as the method of administration for this preliminary experiment. Lung toxicity observed by us following inhalation of TiO_2_ is consistent with observations in literature where impaired mouse lung function was seen due to alveolar septal thickening and neutrophil infiltration upon TiO_2_ exposure^68^. PLGA nanoparticles, on the other hand, did not cause any toxicity which agrees with earlier reports where inhalation of PLGA nanoparticles elicited the lowest inflammatory response when compared to non-biodegradable polystyrene nanoparticles^69^. Rat lungs following treatment with MDNPs looked similar to lungs treated with PLGA nanoparticles and 0.9% saline indicating that our nanoparticle formulation is non-toxic.

There are, however, a few limitations associated with our studies. First of all, the comparatively slower tumor growth rate in the ‘empty MDNPs’ group compared to the sham control group could be due to possible release and subsequent toxicity of SPIO from the particles. SPIO was only used as a proof-of-concept to show that contrast agents can be incorporated within MDNPs. This limitation can be overcome by replacing SPIO with FDA-approved Feraheme or other MR contrast agents such as manganese or Gd, as needed in the future. Another limitation is the use of mice with lung tumors implanted in the hindlimb for our *in vivo* therapeutic studies as this model does not closely mimic the true nature of lung tumors. Our preliminary *in vivo* studies demonstrated here as a proof of concept show the potential use of our nanoparticle system for lung cancer therapy. Future studies will focus on MDNP delivery via nebulization to the orthotopically developed lung tumors in rodents as this delivery strategy and animal model will resemble clinical trials more closely.

## Conclusions

To summarize, novel multi-functional core-shell NPs for combined therapy - chemo-radiotherapy, were synthesized to effectively treat lung cancers. The biodegradable, stimuli-sensitive MDNPs were cyto- and hemo-compatible *in vitro* and demonstrated dose-dependent caveolae-mediated uptake. *In vivo* studies confirmed that our particles can be visualized by MRI and demonstrated effective therapeutic efficacy by slowing tumor growth when administering drug-loaded MDNPs in combination with radiation treatment. The particles did not show significant lung toxicity on nebulization. Our results thus demonstrate the potential of MDNPs, which could be used for simultaneous radiotherapy and chemotherapy, for effective lung cancer treatment. This nanoparticle platform utilizes nanomedicine and drug delivery approaches for controlled dual release of therapeutic compounds to effectively inhibit/ablate lung tumors.

## Methods

### Preparation of MDNPs

The PLGA core was prepared by a standard emulsion method, as previously described^41^. Briefly, 4.5 mg NU7441, 20 mg SPIO, and 90 mg PLGA (L/G ratio: 50:50, inherent viscosity: 0.15–0.25 dl/g) in 5 ml dichloromethane solution was added dropwise to 5% (w/v) PVA (MW: 13,000–23,000) solution and sonicated for 10 minutes at 50 W. Following overnight stirring, the solution was centrifuged at 15,000 rpm for 30 minutes, washed, and lyophilized to obtain the PLGA NPs. The supernatant obtained after the centrifugation step was collected and stored to determine the un-loaded NU7441 amount, which was then used to calculate the loading efficiency of the PLGA core using Equation 1.

1 $$Loading\,efficiency\,( \% )=\frac{(Total\,amount\,of\,drug\,used-Unloaded\,drug)}{Total\,amount\,of\,drug\,used}\times 100 \% $$

The obtained PLGA NPs were surface-functionalized with Allylamine (AH) by carbodiimide chemistry as described previously^70^, during which the carboxyl groups on PLGA were activated and crosslinked to amine groups on AH. The PNIPAAm-CMC polymerization was then performed to provide a shell to the final NP system. Briefly, 58 mg of NIPA, 6 mg of CMC, 13 mg of N,N′-methylenebisacrylamide (BIS) and 50 mg of Sodium dodecyl sulfate (SDS) were added consecutively to 28 mg of PLGA-AH particles in 100 μl DI water during sonication. 0.08% (w/v) of Ammonium Persulfate (APS) and 50 μl of TEMED was added to this solution and the reaction was allowed to continue in Nitrogen gas for 4–6 hours. Folic acid was further conjugated onto the core-shell particles by carbodiimide chemistry as described previously^71^. Briefly, 0.1% w/v of folic acid was added to 5 ml of 2-(N-morpholino)ethanesulfonic acid (MES) buffer and 20 mg each of N-(3-dimethylaminopropyl)-N′-ethylcarbodiimidehydrochloride (EDC) and N-hydroxysuccinimide (NHS) were added 30 minutes apart while keeping the solution shaking at room temperature. Finally, 5 mg of the core-shell particles was added, the suspension was sonicated for 2 minutes at 20 W and then incubated at room temperature overnight. Folic acid-conjugated particles were obtained by centrifugation at 15,000 rpm for 20 minutes.

To load gemcitabine hydrochloride, 5 mg of the MDNPs were incubated with 1 mg of gemcitabine hydrochloride and kept shaking at 4 °C for 3 days to allow drug diffusion into the particles. Since 4 °C is below the lower critical solution temperature (LCST) of PNIPAAm-CMC, the shell is expected to swell due to hydrogen bonding between amide groups in PNIPA and water molecules, and to take in the gemcitabine hydrochloride solution. When the surrounding temperature is raised to above the LCST (43 °C), the PNIPAAm-CMC would shrink and release the encapsulated gemcitabine^72,73^. The final particles were collected by centrifugation and lyophilization. The supernatant following centrifugation was collected and analyzed to determine the amount of un-loaded gemcitabine, which was further used to determine loading efficiency of the particles using equation 1.

## Characterization of MDNPs

### Physical Properties

The MDNP size, polydispersity, and zeta potential values were obtained using dynamic light scattering (DLS, ZetaPals, Brookhaven Instrument, Holtsville, NY) while TEM (TEM, FEI Tecnai G2 Spirit BioTWIN, Hillsboro, OR) was used to observe the size and morphology of the particles. In order to confirm incorporation of all components used during synthesis, Fourier-Transform Infrared Spectroscopy (FTIR) was also conducted on individual components as well as at different stages of nanoparticle preparation.

### Stimuli-Responsive Properties

To study pH-responsiveness of MDNPs, particle size measurements were obtained by DLS following their dispersion in solutions of varying pH at room temperature. To determine the LCST, 5 mg/ml MDNP suspension was placed in a quartz cuvette (Starna Cells, Atascadero, CA) and submerged in a transparent water tank in which the temperature was varied. The scattering of laser light (609 nm) by the MDNPs was captured by a photomultiplier (PMT) at a 90 degree angle. A 594 nm long-pass filter was used as the captured-light filter. The signal was averaged 100 times following which the peak intensity of the emission decay curve was used to calculate the intensity. Photographs of the nanoparticle suspension were also taken before and at the LCST to observe the cloudiness of the polymer network.

### Stability, Drug Release, and Degradation Characteristics

To determine stability, MDNP sizes were studied on incubation with 10% FBS, saline, and Gamble’s solution (simulated human lung fluid) at body temperature (37 °C) using DLS over a period of 5 days. For drug release studies, the MDNP suspensions were subjected to either 37 °C and pH 7.4, or 37 °C and pH 6 conditions. At pre-determined time points, the particles were collected using an external magnet (1.3 Tesla) and the supernatant saved for analysis. Gemcitabine release was quantified at 234 nm absorbance, while NU7441 release was detected at λex 470 nm and λem 520 nm using a spectrophotometer. Furthermore, degradation studies were conducted in which MDNPs placed at 37 °C in PBS, and the particle weight loss over a time range was then measured.

## *In vitro* Cell Studies of MDNPs

### Cytotoxicity Studies

Human dermal fibroblasts (HDFs) and Alveolar Type 1 epithelial cells (AT1) were seeded at a density of 5000 cells/well in a 96 well plate and allowed to be confluent. Then the cells were incubated with varying concentrations of MDNPs (0 [control], 100, 250, 500, 1000, and 2000 µg/ml) for 24 hours. Following incubation, cell viability was assessed using MTS cell viability assays (CellTiter 96®AQueous One Solution Cell Proliferation Assay, Promega, Madison, WI) and validated using Picogreen dsDNA assays (Life Technologies, Grand Island, NY) according to manufacturers’ instructions.

### Cellular Uptake Studies

Prior to cellular uptake studies, the expression of folate receptors on A549 and H460 lung cancer cells was determined using Western blot and Resonant Sensors Bioassay system (Supplementary section 1.1, 1.2). To study *in vitro* cellular particle uptake, A549 and H460 lung cancer cells (seeding density: 5000 cells/well in 96 well plates) were incubated with MDNPs at different concentrations (0, 100, 200, 300, and 500 µg/ml) at 37 °C for 2 hours. For the mechanism of uptake study, cells were exposed to different endocytic inhibitors –10 μg/ml chlorpromazine to inhibit clathrin-dependent endocytosis, 1 μg/ml filipin III to inhibit caveloae-dependent endocytosis, and 50 μM amiloride to inhibit micropinocytosis. Following a one hour incubation with these inhibitors, the cells were washed and treated with 500 µg/ml MDNPs for two hours as the saturation of cellular uptake had occurred in A549 and H460 cells at this condition. At the end of the uptake studies, cells were washed three times with PBS and lysed using 1% Triton X-100. The contents in each well were analyzed using iron assays to detect the amount of NPs internalized by the cells as previously described^74^. This was normalized against the amount of total cell protein per well, determined using BCA assays.

### *In vitro* Clonogenic Assays

In order to study the effects of MDNPs on *in vitro* colony formation, A549 and H460 cells were first seeded in 60 mm petri dishes, and *in vitro* clonogenic assays were performed similar to the procedure described previously^26^. Three treatment groups were used for this study- media control, empty MDNPs, and MDNPs encapsulating NU7441 and gemcitabine. Following cell seeding, the cells were treated with the respective suspensions. Then the dishes were placed at 37 °C undisturbed for 10 days. At the end of the 10-day time-point, the cells were washed well with PBS, fixed and stained using crystal violet staining. The number of colonies in each dish was counted using a light microscope.

### Hemocompatibility Studies

A hemolysis study was conducted as described previously^75^. Human blood from donors was collected and handled following methods approved by the Institutional Review Board at the University of Texas at Arlington and in accordance with relevant guidelines and regulations. Informed consent was obtained from the study participants for participation in the hemocompatibility studies and publication of the results and images. After collection, human blood was incubated with MDNPs at varying concentrations (0, 100, 200, 300, and 500 µg/ml), 0.9% saline (negative control), or distilled water (positive control) for 2 hours. Following centrifugation at 1000 g, absorbance readings were taken at 545 nm. The percentage of hemolysis was calculated using the equation below:

2 $$ \% \,hemolysis=\frac{(Sample\,OD-Negative\,control\,OD)}{(Positive\,control\,OD-Negative\,control\,OD)}\times 100 \% $$

To study blood clotting kinetics, MDNPs of varying concentrations (0 [control], 100, 200, 300, and 500 µg/ml) were added to blood activated with CaCl_2_ and incubated at room temperature (n=9). At pre-determined time points (10, 20, 30, and 60 minutes), the red blood cells (RBCs) not involved in clot formation were lysed. Absorbance readings of supernatant were taken at 540 nm. The absorbance readings were inversely proportional to clot formation. The blood clotting kinetics were also observed visually.

### Preliminary *In vivo* Investigation of MDNPs

#### *In vivo* Imaging

All animal procedures were conducted following the National Institutes of Health’s guidelines and approved by the Institutional Animal Care and Use Committee (IACUC) at the University of Texas (UT) at Arlington and UT Southwestern Medical Center (UTSW) at Dallas. For our preliminary *in vivo* study, H460 tumors were induced in the hind limbs of athymic nude mice, which are commonly used for preliminary *in vivo* assessment of therapeutic efficacy of developed compounds and drug carriers. The animals were monitored in terms of their body weight and tumor volume. When the tumor volumes reached about 100 mm^3^, they were imaged using non-invasive MRI with a 7 T Varian small animal scanner. Multi-echo multi-slice T2 images (TR = 2500 ms; TE = 40 ms; FOV = 40 mm × 40 mm; matrix = 256 × 256; slice thickness = 1 mm) were obtained. Then the animals were randomly assigned to different groups: control, unconjugated MDNPs (1 mg), or folic acid-conjugated MDNPs (1 mg). Twenty-four hours post particle injection, the animals were imaged again using MRI. Then the animals were sacrificed, and Prussian blue staining was done on the tumor sections to detect the presence of iron oxide embedded in the MDNPs.

#### *In vivo* Therapeutic Efficacy

H460 tumors were induced in the hind limbs of athymic nude mice as described above. Once the tumor reached 100 mm^3^ volume, the mice were randomly assigned to the different groups: Sham control, Radiation only, Drug cocktail (NU7741 and gemcitabine), Drug cocktail + Radiation, empty MDNPs, and MDNPs with drugs (NU7741 and gemcitabine) + Radiation. All animals were anesthetized by 1% isoflurane inhalation following which they were administered with the same solutions on alternate days for 2 weeks. Tumors were treated with a radiation dose of 2 Gy/day for 5 days using X-RAD320 small animal irradiator (Precision X-Ray, North Branford, CT) 24 hours following intra-tumoral administration of the drug-containing MDNPs. Tumor volumes and body weights were measured and recorded prior to the injections at each time point. The data obtained was plotted as % increase in tumor volume compared to the tumor volume on day 0 of the treatment experiment.

#### Histopathological Assessment of MDNP Toxicity *in vivo*

Sprague Dawley rats of both sexes (250–400 grams body weight, Charles River Laboratories) were selected for MDNP toxicity assessment as this animal model provided a large surface area for inhalation of MDNPs via a nebulizer (Aeroneb lab nebulizer, Kent Scientific Corporation) to accurately determine the particle toxicity on the lung and other organs. Animals were anesthetized by an intraperitoneal injection of 50 mg/kg ketamine and 5 mg/kg xylazine. The rats were then intubated using a 14 gauge cannula inserted directly into the trachea. Post intubation, 300 µL of either negative control (i.e. 0.9% saline), bare PLGA NPs (1 mg/mL), bare MDNPs (1 mg/mL) or positive control (i.e. titanium dioxide at 1 mg/mL, TiO_2_) were administered to the rats (n = 3 per group) via nebulization through the tracheal cannula. Following nebulization, the rats were observed until complete recovery from the procedures. Seven days after treatment, the rats were sacrificed and their lungs extracted intact and inflated by tracheal instillation of 4% paraformaldehyde at an airway pressure of 25 cm H_2_O. Additionally, other organs namely the heart, liver, kidneys and spleen were collected and fixed in 4% paraformaldehyde. Tissue specimens were then embedded in paraffin, sectioned and stained with hematoxylin and eosin stain.

### Statistical Analysis

The sample size for all the conducted experiments was four (n = 4) if not specified. Statistical analysis on the data was carried out using two-way analysis of variance (ANOVA), and post hoc comparisons (StatView V 5.0.1, SAS Institute Inc., Cary, NC). Statistical significance was determined when p < 0.05.

### Data Availability

The datasets generated and analyzed during the current study are available from the corresponding author on reasonable request.

## Electronic supplementary material

Supplementary Information
