# Supplementary material for: Dual-Drug Containing Core-Shell Nanoparticles for Lung Cancer Therapy
Source: Sci Rep. 2017 Oct 16;7:13249. doi: 10.1038/s41598-017-13320-4 (PMC5643549; doi:10.1038/s41598-017-13320-4)
Supplement: Supplementary file 1 — Supplementary Information [file 41598_2017_13320_MOESM1_ESM.pdf]

Dual-Drug Containing Core-Shell Nanoparticles for Lung Cancer Therapy

Jyothi U. Menon,[1,2] Aneetta Kuriakose,[1,2] Roshni Iyer, [1,2] Elizabeth Hernandez,[3] Leah Gandee,[3] Shanrong Zhang,[4] Masaya Takahashi,[4] Zhang Zhang,[5,6] Debabrata Saha,[5,6*] Kytai T. Nguyen[1,2*]

[1]Bioengineering Department, University of Texas at Arlington, Arlington, TX, 76019 USA

[2]Graduate Biomedical Engineering Program, [3]Department of Urology, [4]Advanced Imaging Research Center, [5]Department of Radiation Oncology, and [6]Simmons Comprehensive Cancer Center at UT Southwestern Medical Center, Dallas, TX, 75390 USA

Corresponding Authors
* Dr. Kytai T. Nguyen
Department of Bioengineering
University of Texas at Arlington
500 UTA Blvd, ERB 241, Arlington, TX 76019 USA
Email: knguyen@uta.edu
TEL: +1-817-272-2540
FAX: +1-817-272-2251

*Dr. Debabrata Saha
Department of Radiation Oncology
Division of Molecular Radiation Biology
University of Texas Southwestern Medical Center
2201 Inwood Road, Dallas, TX 75390 USA
Email: debabrata.saha@utsouthwestern.edu
Tel: +1-214-648-7750
FAX: +1-214-648-5995

Supplementary Materials

1. Materials and Methods

1.1. Western Blot Analysis of Folic Acid Receptors on Lung Cancer Cells

Two different lung cancer cells, A549 and H460, were studied for folate receptor expression. The cells were lysed, and the total cell protein was quantified using the Bradford assay (Bio-Rad, Hercules, CA), as described previously [1], and probed with Anti-folate binding protein antibodies (Abcam, Cambridge, MA) per manufacturer's instructions.

1.2. Analysis of Binding Efficiency of Folic Acid to Cellular Folate Receptors

The binding of folic acid to folate receptors was studied using the Resonant Sensors Bioassay system (Resonant Sensors Inc. (RSI), Arlington, TX). A549 and H460 lung cancer cells, as well as Alveolar Type-1 cells (AT1), were seeded at a density of 10,000 cells/well in RSI sensor 96-well plates, which were then incubated at 37°C for 24 hours to allow for cell attachment. The cells were then incubated with folic acid at varying concentrations (0, 0.1, 1, 5, 10, and 15 µM) for 3 hours in the RSI system and peak shift with cell binding over time was studied. The results were plotted as peak shift vs. time where greater binding of folic acid to cells resulted in greater peak shifts.

1.3. Magnetic Properties of MDNPs

In order to determine iron content within the particles, an iron assay was conducted as described previously [2]. Magnetic property of the MDNPs was also analyzed using a SQUID magnetometer as described before [3]. Finally, agarose phantoms (0.5% w/v) containing varying

MDNP concentrations (0, 0.25, 0.5, 1, 2 mg/ml) were prepared and imaged using MRI. All images were conducted on a 7T Agilent (Varian) MRI Scanner with a fast spin-echo sequence. Some major parameters were as follows: repetition time (TR) =5000 ms, echo time (TE) = 8.58 ms, FOV = 35 x 35 mm, slice thickness = 1 mm.

1.4. Cell Activation Studies

To detect the intracellular Reactive Oxygen Species (ROS) production, AT1 cells were incubated with MDNPs for 24 hours and then washed three times with PBS and incubated with 5μM of 2’, 7’-dichlorodihydrofluorescein diacetate (H2DCFDA) solution in PBS for 30 minutes. The cells were then washed again and fluorescence was measured at λex of 485 nm and λex of 530 nm using a fluorospectrophotometer. To study cytokine production after particle exposure, interleukins 6, 8, 10 and tumor necrosis factor-alpha (TNF-α) in the medium were measured using Multi-Analyte ELISA Array Kits (SA Biosciences, Frederick, MD) according to the manufacturer's directions.

2. Tables

Table 1. Size, surface charge, and polydispersity values of the NPs at different stages of preparation.

	**Diameter (nm)**	**Polydispersity**	**Zeta potential (mV)**
**PLGA-SPIO NPs**	230 ± 98	0.22 ± 0.40	-12
**AH-modified PLGA-SPIO NPs**	262 ± 79	0.12 ± 0.23	-18
**MDNPs**	289 ± 49	0.32 ± 0.31	-36

3. Results

3.1. Other Characterization Results of MDNPs

The successful incorporation of all components within the MDNPs was confirmed using FTIR as shown in **Figure S1A**. The OH stretching characteristic of COOH groups in PLGA can be seen in PLGA-MNPs as well as AH-modified PLGA-MNPs between 3200–3300 cm^{-1}. The incorporation of SPIO was confirmed at all stages of synthesis by the distinctive peak observed between 530 to 570 cm^{-1}. Furthermore, the peaks at 1720 cm^{-1} for PLGA-MNPs and 1750 cm^{-1} for AH represent the carbonyl groups seen in ester linkages within PLGA. The FTIR spectra for AH-modified PLGA-MNPs also shows a distinct peak at 1560 cm^{-1} for $C{=}ONH_2$ which represents successful coupling of Allylamine with the PLGA surface. The amide peaks at around 1670 cm^{-1} for both MDNPs and folic acid-conjugated MDNPs are characteristics for both chitosan and PNIPAAm. Furthermore, peaks at around 1590 cm^{-1} indicate asymmetric stretching vibrations of COO^- due to the presence of carboxymethyl groups on the chitosan component. CH_2 peaks of PNIPAAm are also visible at 2920 cm^{-1}. Folic acid conjugation was confirmed by the presence of an aromatic ring stretch at 1430 cm^{-1} and the amide bonds at 1670 and 1650 cm^{-1}.

Particle size measurements over a period of 5 days indicated that the MDNPs maintained their size and did not aggregate in 10% FBS, 0.9% saline, and Gamble's solution at 37°C (**Figure S1B**). Furthermore, degradation studies showed that there was a 24% decrease in MDNP weight in 5 days. By day 27, there was a 63% decrease in the weight of the MDNPs compared to their initial weight (**Figure S1C**). In order to confirm stimuli-sensitive properties of MDNPs, two studies were performed. A significant decrease in particle size was observed from pH 6 to pH 5 indicating that the particles shrink in response to an acidic pH environment (**Figure S1D**). LCST measurements confirmed that PNIPAAm-CMC can undergo a rapid, reversible phase

transition at an LCST of 43°C. A distinct cloudiness in the PNIPAAm-CMC NP suspension was also observed at around 43°C, while it remained clear below this temperature (**Figure S1E**). LCST of the particles was not affected by changes in pH.

The iron content of MDNPs was observed to be about 47%. Visual observation and SQUID magnetometry were used to establish that the MDNPs retained the superparamagnetic behavior. MDNPs dispersed in DI water could rapidly move in the direction of the applied 1.3T magnet as evident from **Figure S1F** (inset). The hysteresis loop for MDNPs showed that the particles retained their magnetic properties with a remanence of 6.45 (M_r/M_s) and coercivity of 52.6 Oe. Bare SPIO showed a remanence of 7.16 (M_r/M_s) and coercivity of 66.7 Oe. Furthermore, T2-weighted MR images of MDNPs-containing agarose phantoms showed darker negative contrast with increasing MDNP concentration (**Figure S1G**). It was observed that there was almost an 87% decrease in signal intensity in the case of agarose phantoms containing 2 mg/ml MDNP, while 0.25 mg/ml, 0.5 mg/ml, and 1 mg/ml MDNP concentrations caused the signal intensity to drop by 36%, 55%, and 75% (data not shown), respectively, compared to the control (agarose phantom only).

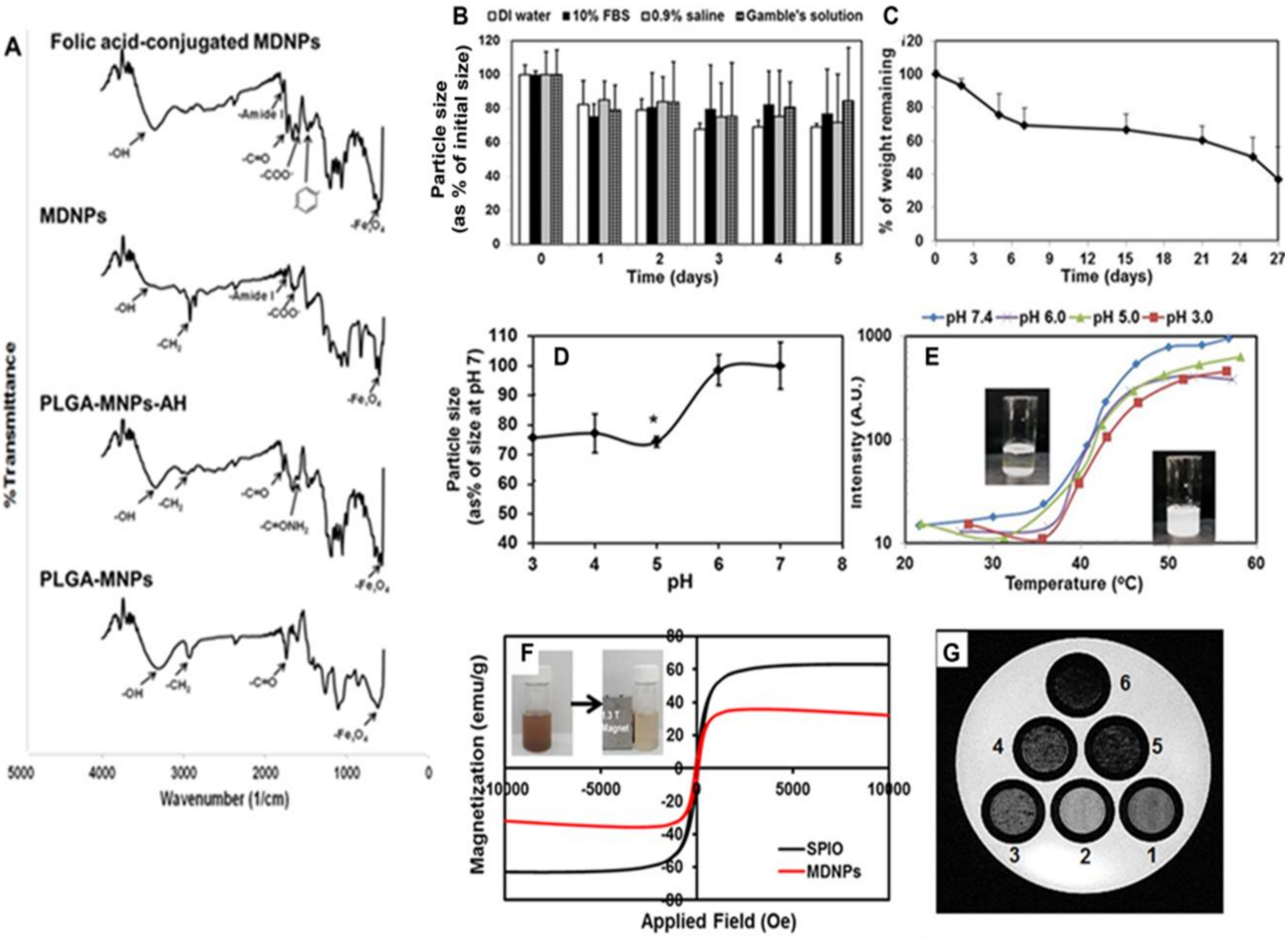

Figure S1: Characterization of MDNPs. **(A)** FTIR spectra of each step of nanoparticle synthesis demonstrating that all the components have been incorporated in the final nanoparticle system. **(B)** Stability studies indicating minimal MDNP size variations on incubation in 10% serum, 0.9% saline, and Gamble's solution over 5 days. **(C)** Degradation studies showing a decrease in MDNP weight by 63% in 4 weeks. (**D**) Significant decrease in MDNP size observed with change in environmental pH from 7.4 to 6 (n=4, *p<0.05). **(E)** MDNPs demonstrated LCST of 43°C with no significant LCST variation with pH. LCST changes were observed visually (*inset*). **(F)** Hysteresis loop indicating superparamagnetic property of MDNPs. Magnetic behavior of MDNPs in response to application of 1.3 T magnet was observed visually (inset). **(G)** MR images of agarose phantoms containing (1) Agarose only, (2) MDNPs without iron oxide, and MDNPs at (3) 0.25 mg/ml, (4) 0.5 mg/ml, (5) 1 mg/ml, and (6) 2 mg/ml concentration

3.2. Western Blot and Folic Acid Binding Efficiency

Western blot results demonstrated that the folate receptors were overexpressed on both A549 and H460 lung cancer cells (**Figure S2A**). Our studies with the RSI instrument indicated minimal peak shift on incubating AT1 cells with folic acid (**Figure S2B**). This peak shift was also not dependent on the concentration of folic acid. On the other hand, H460 cells had comparatively larger peak shifts than both A549 and ATI cells (**Figures S2B-D**). The larger peak shifts of H460 compared to A549 and AT1 cells indicate that H460 cells have more affinity for binding to folic acid.

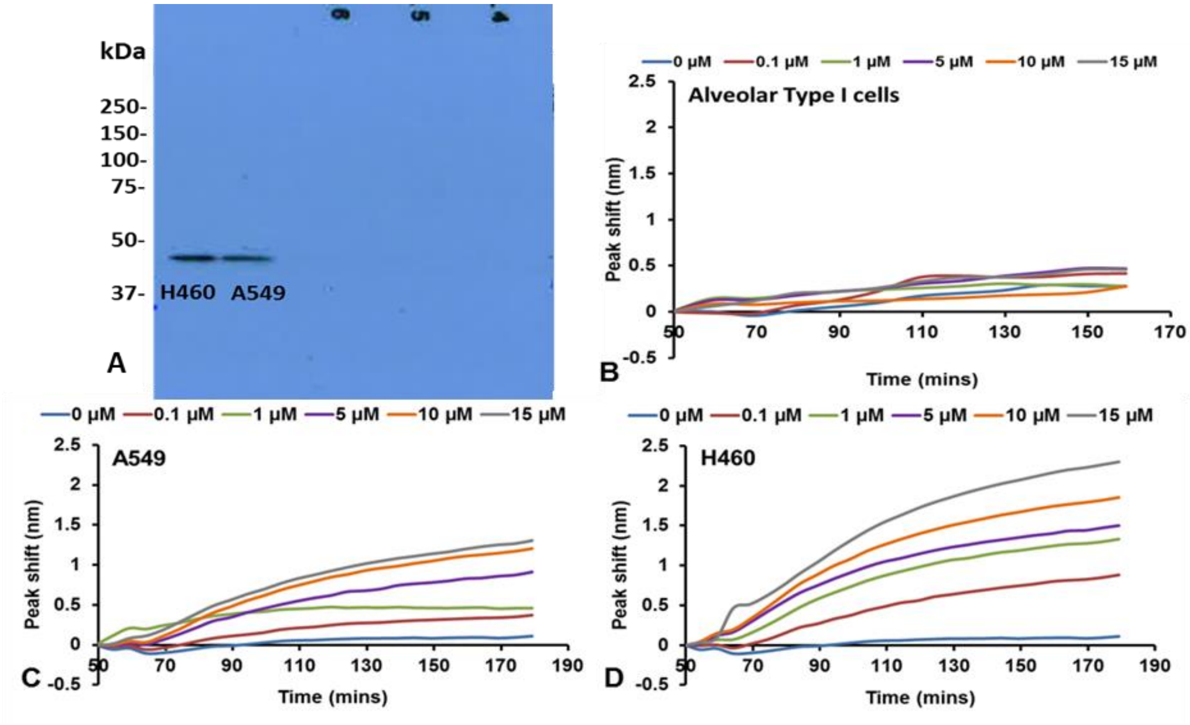

Figure S2: (A) Western blot results indicating the overexpression of folate α receptors on both H460 and A549 lung cancer cells. RSI bioassay system results demonstrating that **(B)** there is no dose-dependent increase in peak shift on treatment of human alveolar type I cells with folic acid. An increase in peak shift was observed for both **(C)** A549 and **(D)** H460 cells compared to their respective controls (0 μM) indicating folic acid binding to cell surface receptors. H460 showed a comparatively larger peak shift indicating greater affinity for folic acid (n=4).

3.3. *Other In vitro Experiments using MDNPs*

Cytotoxicity studies: *In vitro* cytotoxicity studies conducted using Picogreen dsDNA assays demonstrated that >80% of DNA content in each well was retained up to 1 mg/ml MDNP concentration. However, a decrease in DNA content was observed in samples treated with 2 mg/ml MDNP concentration. (**Figure S3A**). These results are in agreement with the results obtained using MTS assays, and confirm that the MDNPs are cytocompatible *in vitro* with HDFs and AT2 cells up to a concentration of 1 mg/ml.

Cellular uptake studies using normal lung cells: Our results demonstrated that for AT1 cells and Human bronchial epithelial cells (HBECs), there is a dose-dependent uptake of MDNPs (**Figure S3B**). However, this uptake (< 0.9 μg iron/ μg cell protein in healthy cells on treatment with 500 μg/ml MDNPs) is less than what was observed in the case of A549 and H460 cells (>1.6 μg iron/ μg cell protein on treatment with 500 μg/ml MDNPs).

Cell Activation studies: Previous studies have shown that exposure to toxic particles resulted in excess ROS production in the lungs followed by the production of inflammatory cytokines, eventually leading to inflammation and fibrosis [4]. Therefore, production of these molecules was tested to ensure that the MDNPs were not toxic when in contact with lung cells following administration. Cell activation studies were conducted by incubating MDNPs with AT1 cells for 24 hours. Following this, cytokine and ROS production from the exposed cells were analyzed. The cells stimulated with LPS (positive control) released 0.25 ng/ml, 1.27 ng/ml, 0.24 ng/ml, and 0.11 ng/ml of IL-6, IL-8, IL-10, and TNF-α inflammatory cytokines, respectively. In comparison, MDNPs showed a significantly lower amount of IL-6, IL-8, and IL-10 production and negligible amounts of TNF-α was released (**Figure S3C**). Cells exposed to 1

mg/ml MDNP concentration produced only 0.07 ng/ml IL-6, 0.73 ng/ml IL-8, 0.05 ng/ml IL-10, and 0.02 ng/ml TNF-α, which was significantly lower than the amounts produced by LPS stimulation. Similarly, ROS produced by the cells treated with 1 mg/ml MDNPs was only 16% of that produced by the cells treated with LPS (**Figure S3D**). These results indicate that exposure of cells to MDNPs does not cause significant cell activation or initiate significant inflammatory reactions in healthy lung cells.

.

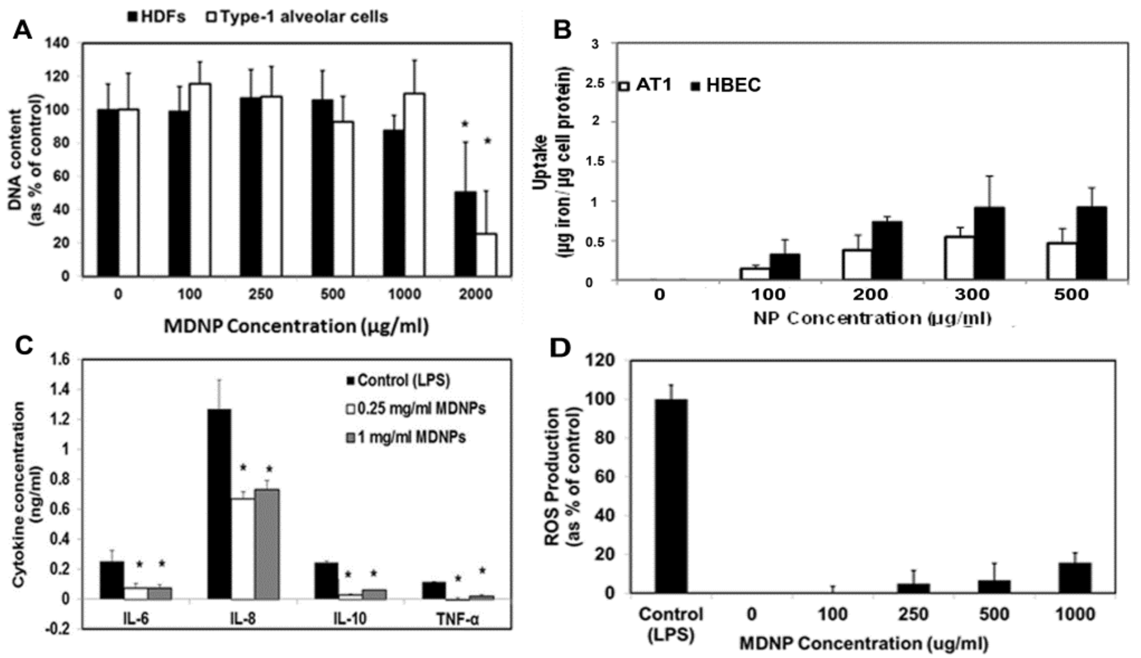

Figure S3: Other *in vitro* studies using MDNPs. (**A**) *In vitro* MDNP cytocompatibility studies on HDFs and alveolar Type 1 cells using Picogreen dsDNA assay also demonstrated that cells were viable up to a high concentration of 1 mg/ml (n=4, *p<0.05). (**B**) *In vitro* cellular uptake of MDNPs by Alveolar Type I cells and Human bronchial epithelial cells (HBECs) demonstrating dose-dependent uptake of MDNPs up to 200 μg/ml MDNP concentration (n=4, *p<0.05). **(C)** Inflammatory cytokine release from cells following 24-hour exposure to MDNPs at two concentrations: 0.25 mg/ml and 1 mg/ml. Significantly smaller quantities of IL-6, IL-8, IL-10, and TNF-α were produced compared to the positive control (LPS stimulated cells) (n=4, *p<0.05 w.r.t control). **(D)** ROS production from cells was found to be about 16% compared to LPS control even at a high MDNP concentration of 1000 μg/ml.

3.5. Visual observation of samples in hemocompatibility studies

Clear reddening of the solution was observed for the positive control in the hemolysis study, which indicated hemolysis. No visual indication of hemolysis was seen in the negative control and groups treated with nanoparticles (blood exposed to 100, 200, 300, and 500 µg/ml MDNP concentration) (**Figure S4A**). Visual observation of samples from blood clotting studies indicated that exposure to MDNPs did not result in any changes in blood clot formation (**Figure S4B**).

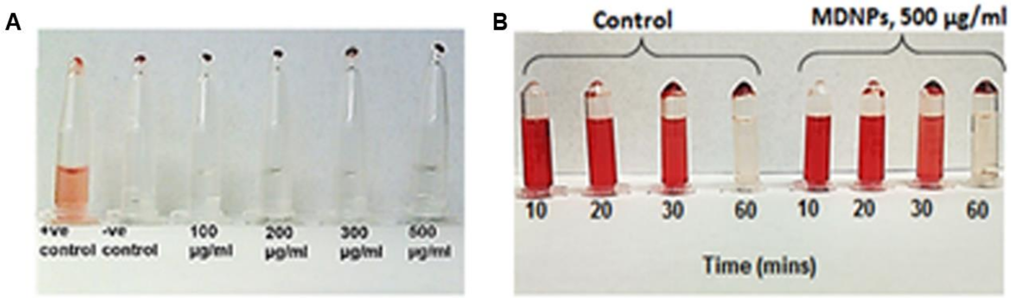

Figure S4: (A) Visual observation confirmed that minimal hemolysis occurred in negative control and experimental groups while distinct reddishness was seen in the positive control. (B) Visual observation of the blood clotting in control group and 500 µg/ml MDNP group (n=9).

References

1. Kong, Z., et al., *Downregulation of human DAB2IP gene expression in prostate cancer cells results in resistance to ionizing radiation.* Cancer Res, 2010. **70**(7): p. 2829-2839.
2. Rahimi, M., et al., *In vitro evaluation of novel polymer-coated magnetic nanoparticles for controlled drug delivery.* Nanomed Nanotech Biol Med, 2010. **6**(5): p. 672-680.
3. Wadajkar, A.S., et al., *Multifunctional particles for melanoma-targeted drug delivery.* Acta Biomater, 2012. **8**(8): p. 2996-3004.
4. Nel, A., et al., *Toxic potential of materials at the nanolevel.* Science, 2006. **311**(5761): p. 622-627.
